# AGCLD: an adaptive graph contrastive learning method with denoising for spatial domain identification

**DOI:** 10.1093/bib/bbag385

**Published:** 2026-07-14

**Authors:** Yating Li, Xinyue Yu, Hao Zhang, Hao Lin, Bo Liu, Haixia Long

**Affiliations:** School of Artificial Intelligence, Hainan Normal University, No. 99 Longkun South Road, Qiongshan District, Haikou 571158, Hainan, China; Key Laboratory of Data Science and Smart Education, Ministry of Education, Hainan Normal University, No. 99 Longkun South Road, Qiongshan District, Haikou 571158, Hainan, China; Haikou Key Laboratory of Intelligent Analysis and Secure Sharing of Tropical Biodiversity Data, Hainan Normal University, No. 99 Longkun South Road, Qiongshan District, Haikou 571158, Hainan, China; School of Artificial Intelligence, Hainan Normal University, No. 99 Longkun South Road, Qiongshan District, Haikou 571158, Hainan, China; Key Laboratory of Data Science and Smart Education, Ministry of Education, Hainan Normal University, No. 99 Longkun South Road, Qiongshan District, Haikou 571158, Hainan, China; Haikou Key Laboratory of Intelligent Analysis and Secure Sharing of Tropical Biodiversity Data, Hainan Normal University, No. 99 Longkun South Road, Qiongshan District, Haikou 571158, Hainan, China; School of Artificial Intelligence, Hainan Normal University, No. 99 Longkun South Road, Qiongshan District, Haikou 571158, Hainan, China; Key Laboratory of Data Science and Smart Education, Ministry of Education, Hainan Normal University, No. 99 Longkun South Road, Qiongshan District, Haikou 571158, Hainan, China; Haikou Key Laboratory of Intelligent Analysis and Secure Sharing of Tropical Biodiversity Data, Hainan Normal University, No. 99 Longkun South Road, Qiongshan District, Haikou 571158, Hainan, China; School of Life Science and Technology, University of Electronic Science and Technology of China, No. 4, Section 2, North Jianshe Road, Chengdu 610054, China; School of Artificial Intelligence, Hainan Normal University, No. 99 Longkun South Road, Qiongshan District, Haikou 571158, Hainan, China; Key Laboratory of Data Science and Smart Education, Ministry of Education, Hainan Normal University, No. 99 Longkun South Road, Qiongshan District, Haikou 571158, Hainan, China; Haikou Key Laboratory of Intelligent Analysis and Secure Sharing of Tropical Biodiversity Data, Hainan Normal University, No. 99 Longkun South Road, Qiongshan District, Haikou 571158, Hainan, China; School of Artificial Intelligence, Hainan Normal University, No. 99 Longkun South Road, Qiongshan District, Haikou 571158, Hainan, China; Key Laboratory of Data Science and Smart Education, Ministry of Education, Hainan Normal University, No. 99 Longkun South Road, Qiongshan District, Haikou 571158, Hainan, China; Haikou Key Laboratory of Intelligent Analysis and Secure Sharing of Tropical Biodiversity Data, Hainan Normal University, No. 99 Longkun South Road, Qiongshan District, Haikou 571158, Hainan, China

**Keywords:** single-cell, spatial multi-omics, adaptive graph, multi-head self-attention, contrastive learning

## Abstract

Single-cell spatial multi-omics technologies enable the simultaneous acquisition of multimodal molecular profiles and spatial location information in situ, providing a novel perspective for spatial domain identification and functional characterization of tissues. However, existing methods still suffer from several limitations, including insufficient denoising capability for single-cell data, reliance on static graph structures, and inadequate exploitation of the complementary relationships between spatial information and molecular features. To address these challenges, we propose AGCLD, an adaptive graph contrastive learning method with denoising for spatial domain identification. Specifically, a modality-specific denoising variational autoencoder is first employed to learn robust latent representations, thereby effectively mitigating noise interference. A differentiable graph generator is then introduced to adaptively construct spatial adjacency graphs and expression similarity graphs, alleviating the bias introduced by fixed neighborhood assumptions. Finally, AGCLD utilizes a dual-graph attention network to encode the spatial adjacency and expression similarity graphs, yielding spatial and feature embeddings, and incorporates a contrastive learning mechanism to align the dual-view representations, thereby enhancing representation consistency. Extensive experiments on five spatial multi-omics datasets demonstrate that AGCLD outperforms state-of-the-art methods, including SpatialGlue, in spatial domain identification tasks.

## Introduction

Cells are the basic units of life, and spatial domains, as functional regions of cells within tissues, are essential for understanding cellular heterogeneity [1–3]. Accurate identification of spatial domains is therefore a key step in studying tissue organization [4]. The development of single-cell sequencing technologies has overcome the limitation of traditional bulk sequencing, which only captures averaged signals, and enables the analysis of cellular states and functional differences at single-cell resolution [5–7]. However, conventional single-cell sequencing often loses the spatial context of cells in tissues, which limits a deeper understanding of their spatial organization. In recent years, spatial multi-omics technologies, such as DBiT-seq [8], Spatial CITE-seq [9], and spatial ATAC-RNA-seq [10], have been rapidly developed, allowing the simultaneous measurement of multi-omics data together with spatial information and providing new opportunities for studying tissue microenvironments [11–14].

However, due to differences in measurement techniques and biological properties, different types of single-cell omics data often show different noise patterns and sparsity levels [15–17]. For example, proteomics data usually have a relatively high signal-to-noise ratio, while transcriptomics data are highly sparse [18, 19]. Traditional linear denoising methods are not suitable for handling such heterogeneity across modalities, which limits the accuracy and reliability of multi-omics integration [20]. In addition, spatial neighborhood relationships in spatial multi-omics data are often locally heterogeneous [21]. Cells in different regions may have different interaction patterns, making it difficult for models with fixed neighborhood parameters to capture such dynamic spatial structures [22–24]. Moreover, spatial information and molecular features describe the same biological system from different perspectives. Simple concatenation or linear fusion methods cannot fully capture their complementary information and underlying biological relationships [25, 26]. Therefore, there is a strong need for computational methods that can achieve effective denoising, adaptive neighborhood modeling, and reliable cross-modal integration [27–29].

Existing methods for single-cell omics analysis can be roughly divided into two categories. The first category is based on deep generative models, which perform denoising and imputation through latent variable modeling, such as spaVAE [30], SEDR [31], and MultiVI [32]. These methods are effective in handling noisy and sparse data, but they mainly focus on reconstruction and pay less attention to cross-modal integration and spatial relationships. The second category uses graph neural networks or weighted integration strategies to combine multi-source information [33, 34]. For example, WNN [35] constructs a weighted nearest neighbor graph for multimodal integration, and scMDC [36] applies a deep clustering framework to jointly encode concatenated data. For spatial multi-omics data, methods such as SpatialGlue [37], COSMOS [38], and SMOPCA [39] incorporate spatial information into the integration process, but they rely on predefined graph structures and cannot adaptively learn cell-specific neighborhoods. The recently proposed CANDIES [40] method improves integration performance through cross-modal denoising and fusion, but still lacks adaptive modeling of spatial neighborhood structures. Therefore, existing methods commonly suffer from three limitations (insufficient modality-specific denoising, reliance on static graph structures, and limited exploitation of cross-modal complementarity), which prevent accurate spatial domain identification.

To address these limitations in denoising, adaptive graph learning, and multi-omics integration, we propose adaptive graph contrastive learning method with denoising (AGCLD). First, AGCLD uses modality-specific denoising variational autoencoder(DVAE) to model each omics dataset separately, reducing noise while preserving biological information. Then, a differentiable graph generator (DGG) is used to learn adaptive neighborhood structures for each cell and construct spatial adjacency and expression similarity graphs. Finally, the model jointly considers spatial relationships and expression similarity and optimizes cell representations with a contrastive learning objective. This helps to integrate spatial and molecular information and produces low-dimensional embeddings with better consistency and discriminative ability for downstream clustering and spatial domain identification. Experiments on multiple real datasets show that AGCLD outperforms existing methods in clustering accuracy and spatial continuity, demonstrating a strong ability in to capture complex spatial structures.

In summary, the main contributions of this work are as follows:

(1) A denoising variational autoencoder with a masking mechanism is proposed. By randomly masking input features, the model learns robust representations from noisy and sparse data, improving denoising performance and generalization.
(2) A differentiable graph generation mechanism is introduced to construct spatial and feature graphs adaptively. This removes the dependence on predefined graph structures and enables the model to learn neighborhood relationships for each cell.
(3) A dual-graph contrastive learning mechanism is proposed. The spatial and feature representations of the same cell are treated as positive pairs to improve consistency, while representations of different cells are treated as negative pairs to enhance separability. This improves cross-modal alignment and representation quality for spatial domain identification.

## Materials and methods

### Overview of the method

**Figure 1:**
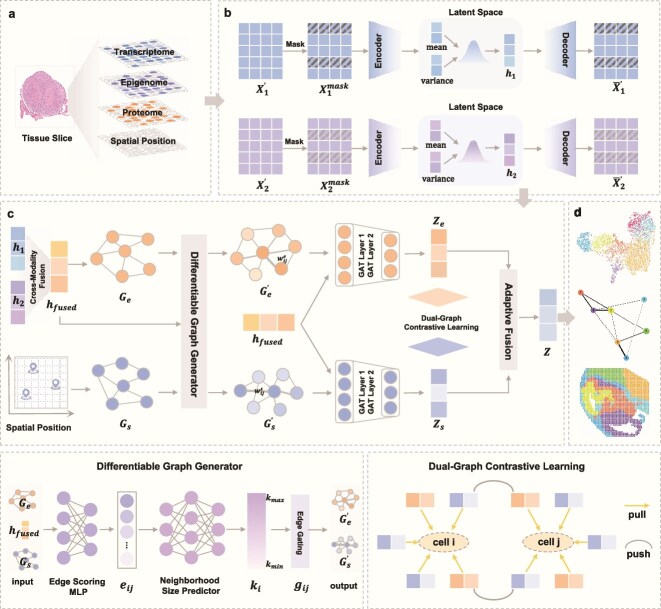
Overall framework of AGCLD.

The overall framework of AGCLD is illustrated in Fig. 1. The single-cell spatial multi-omics dataset typically consists of two omics modalities along with their spatial coordinates. The feature matrices of the two modalities are denoted as $X_{1}$ and $X_{2}$ (Fig. 1a). First, the randomly masked feature matrices $X_{1}^{\textrm{mask}}$ and $X_{2}^{\textrm{mask}}$ are fed into denoising variational autoencoder to learn the latent distributions of each modality, yielding denoised representations $h_{1}$ and $h_{2}$ (Fig. 1b). Based on these representations, AGCLD introduces a multi-head self-attention mechanism to fuse $h_{1}$ and $h_{2}$ across modalities, resulting in a fused representation $h_{\textrm{fused}}$ (Fig. 1c). Next, a spatial adjacency graph $G_{s}$ is constructed according to spatial coordinates, and an expression similarity graph $G_{e}$ is built based on $h_{\textrm{fused}}$. Furthermore, a DGG is employed to adaptively learn edge weights and neighborhood sizes, producing optimized graph structures $G^{\prime}_{s}$ and $G^{\prime}_{e}$. Finally, the fused representation $h_{\textrm{fused}}$ and the optimized graphs are fed into a dual-graph attention encoder to obtain spatial embedding $Z_{s}$ and expression embedding $Z_{e}$. A contrastive learning mechanism is introduced to enhance the consistency between different views. An adaptive fusion module is then applied to obtain a unified embedding $Z$ (Supplementary Fig. S1), which is used for downstream tasks such as spatial domain identification (Fig. 1d). Details of each module are described in the following sections.

### Modality-specific denoising variational autoencoder

Spatial multi-omics data are often affected by factors such as sequencing depth variation and batch effects, resulting in complex nonlinear noise. Although linear dimensionality reduction methods such as principal component analysis (PCA) can be used for preliminary feature compression, they are not sufficient to capture nonlinear structures and complex noise patterns in the data. Therefore, to learn robust representations for each modality, we construct an independent denoising variational autoencoder for each modality [41]. This module takes the preprocessed feature matrices $X^{\prime}_{1}$ and $X^{\prime}_{2}$ as input and produces denoised latent representations $h_{1}$ and $h_{2}$. During training, the input features are randomly masked with probability $p_{\mathrm{mask}}$. The encoder maps the masked input to the parameters of a latent distribution, and the latent representation is obtained via the reparameterization trick. This mechanism encourages the model to recover missing features from the observed (unmasked) information, thereby improving robustness to noise. For the $m$-th modality ($m \in \{1, 2\}$), the masked input $X_{m}^{\mathrm{mask}}$ is encoded to obtain the latent distribution parameters $\mu _{m}$ and $\sigma _{m}$. The latent variable is sampled via reparameterization and then decoded to reconstruct the input: 


(1) \begin{align*} & h_{m} = \mu_{m} + \epsilon \odot \sigma_{m}\end{align*}

(2) \begin{align*}& \bar{X}_{m}^{\prime} = \mathrm{Decoder}(h_{m})\end{align*}

where $\epsilon \sim \mathcal{N}(0, I)$ is a random noise vector sampled from a standard multivariate Gaussian distribution, $h_{m}$ denotes the denoised latent representation, $\mu _{m}$ and $\sigma _{m}$ denote the mean and standard deviation vectors, respectively, and $\bar{X}_{m}$ represents the reconstructed features.

### Cross-modality fusion

Although simple feature concatenation can integrate multimodal information, it is insufficient to capture the interactions between different modalities. To address this, we introduce a multi-head self-attention mechanism to model cross-modal relationships [42, 43]. Specifically, the latent representations $h_{1}$ and $h_{2}$ are first stacked to form a modality sequence $H$. A multi-head self-attention layer is then applied to capture global dependencies across modalities. After that, the enhanced modality features $H_{\mathrm{att}}^{(m)}$ are obtained by aggregating features using attention. To further integrate global context and preserve information specific to each modality, the features enhanced by attention are concatenated with the original modality representations and mapped to a unified fused representation $h_{\mathrm{fused}}$ through a fully connected layer followed by a $\tanh $ activation function. This design improves the consistency between modalities while retaining their complementary characteristics. The computation is defined as follows: 


(3) \begin{align*} & \mathrm{head}_{n} = \mathrm{softmax}\!\left( \frac{(H W_{n}^{Q})(H W_{n}^{K})^{T}}{\sqrt{d_{k}}} \right) (H W_{n}^{V})\end{align*}

(4) \begin{align*}& H_{\mathrm{att}}^{(m)} = \mathrm{Concat}(\mathrm{head}_{1}, \ldots, \mathrm{head}_{r}) W^{O}\end{align*}

(5) \begin{align*}& h_{\mathrm{fused}} = \tanh \left( W_{\mathrm{fuse}} \left[ h_{1} \,\|\, \frac{1}{2} \sum_{m=1}^{2} H_{\mathrm{att}}^{(m)} \,\|\, h_{2} \right] + b_{\mathrm{fuse}} \right)\end{align*}

where $W_{n}^{Q}$, $W_{n}^{K}$, and $W_{n}^{V}$ denote the query, key, and value projection matrices of the $n$-th attention head, respectively. $\mathrm{head}_{n}$ represents the output of the $n$th attention head, with $n = 1, 2, \ldots , r$, $r$ is the number of attention heads. $W^{O}$ is the output projection matrix. $d_{k}$ is the dimensionality of each attention head. $W_{\mathrm{fuse}}$ and $b_{\mathrm{fuse}}$ are the learnable weight matrix and bias term of the fusion layer, respectively.

### Adaptive graph construction

Fixed neighborhood construction strategies are insufficient to capture complex cell–cell relationships in real tissue environments. To address this limitation, we propose a DGG [44], which takes the fused representation $h_{\mathrm{fused}}$ and candidate graphs ($G_{s}$ and $G_{e}$) as input, and outputs optimized graph structures with learned edge weights, denoted as $G^{\prime}_{s} = (V, E_{s}, w^{s}_{ij})$ and $G^{\prime}_{e} = (V, E_{e}, w^{e}_{ij})$. The DGG module consists of two components: edge importance estimation and neighborhood size prediction. The edge scoring network computes edge importance based on node features and pairwise distances, while the degree prediction network estimates the optimal neighborhood size using node features and local topology. Through a gating mechanism and a straight-through estimator, the model enables differentiable edge selection, allowing end-to-end optimization of the graph structure. For each candidate edge $(i,j)$, the DGG adaptively learns edge weights through the following steps: 


(6) \begin{align*} & e_{ij} = \sigma \left( \frac{\mathrm{MLP}_{e} \left( [h_{i} \,\|\, h_{j} \,\|\, d_{ij}] \right) + \mathrm{noise}}{\tau_{e}} \right)\end{align*}

(7) \begin{align*}& k_{i} = k_{\min} + (k_{\max} - k_{\min}) \, \sigma \left( \mathrm{MLP}_{k} \left( h_{i} \,\|\, \sum_{j \in \mathcal{N}(i)} e_{ij} \right) \right)\end{align*}

(8) \begin{align*}& g_{ij} = \sigma \left( \frac{k_{i} - r_{ij}}{\lambda} \right) + \left( \mathbb{I}(r_{ij} \leq k_{i}) - \sigma \left( \frac{k_{i} - r_{ij}}{\lambda} \right) \right) \cdot \mathrm{detach}()\end{align*}

(9) \begin{align*}& w_{ij} = \frac{e_{ij} g_{ij}}{\sum_{k \in \mathcal{N}(i)} e_{ik} g_{ik}}\end{align*}

where $e_{ij}$ denotes the learned importance score of edge $(i,j)$, $d_{ij}$ represents the distance between nodes $i$ and $j$, and $\tau _{e}$ is a temperature parameter controlling the sharpness of edge selection. $k_{i}$ denotes the predicted neighborhood size for node $i$, with $k_{\min }$ and $k_{\max }$ defining its range. $r_{ij}$ represents the rank of node $j$ in the neighborhood of node $i$. $\lambda $ is a scaling parameter for the gating function. $\mathbb{I}(\cdot )$ denotes the indicator function, and $\mathrm{detach}(\cdot )$ is used to stop gradient propagation during backpropagation. $w_{ij}$ denotes the normalized edge weight.

### Dual-graph attention encoder

Spatial proximity and expression similarity characterize cell–cell relationships from structural and functional perspectives, respectively. These two views provide complementary information. Therefore, we construct a dual-graph attention encoder to learn cell representations from both views [45, 46]. Specifically, the fused representation $h_{\mathrm{fused}}$ and the optimized graph structures ($G^{\prime}_{s}$ and $G^{\prime}_{e}$) are used as inputs. For each cell, the model outputs a spatial embedding $Z_{s,i}$ and an expression embedding $Z_{e,i}$. The two branches perform message passing on $G^{\prime}_{s}$ and $G^{\prime}_{e}$, respectively, and aggregate neighborhood information to learn view-specific representations. The spatial branch captures structural information based on spatial coordinates, while the expression branch captures functional information based on feature similarity. Both branches adopt a graph attention network with two layers, where attention coefficients are used to adaptively weight neighbor contributions. The attention coefficients are computed as follows: 


(10) \begin{align*} & Z_{s,i} = \mathrm{GAT}^{(2)}(G^{\prime}_{s}, h_{\mathrm{fused}})_{i}\end{align*}

(11) \begin{align*}& Z_{e,i} = \mathrm{GAT}^{(2)}(G^{\prime}_{e}, h_{\mathrm{fused}})_{i}\end{align*}

where $\mathrm{GAT}^{(2)}(\cdot )$ denotes a two-layer graph attention network.

### Adaptive fusion

To fully utilize the complementary information from the two views and avoid information loss caused by simple averaging, we design an adaptive fusion mechanism. This mechanism dynamically balances the contributions of the spatial and expression views through learnable weights. Specifically, for each cell, the spatial embedding $Z_{s,i}$ and the expression embedding $Z_{e,i}$ are concatenated and fed into a multilayer perceptron. A softmax function with temperature scaling is then applied to compute adaptive weights, and the final unified embedding $Z_{i}$ is obtained as a weighted combination of the two views. This design allows the model to adaptively adjust the importance of each view based on local cellular characteristics. The computation is defined as follows: 


(12) \begin{align*}& Z_{i} = \mathrm{softmax} \left( \frac{\mathrm{MLP}([Z_{s,i} \,\|\, Z_{e,i}])}{\tau} \right) \cdot [Z_{s,i}, Z_{e,i}]^{T}\end{align*}

where $\tau $ is a learnable temperature parameter.

### Training objective

AGCLD is trained in a multi-task learning framework that jointly optimizes five loss components, including Kullback-Leibler (KL) divergence regularization, DVAE reconstruction loss, contrastive loss, DGI loss, and spatial regularization loss. These objectives work together to improve the robustness of the learned representations.The KL divergence is first introduced to regularize the latent distribution. It constrains the learned distribution to be close to a standard normal distribution by penalizing deviations of the mean from zero and the variance from one. This helps to maintain a latent space with clear structure and prevent distribution collapse, while encouraging smooth interpolation in the latent space. The KL divergence loss is defined as follows: 


(13) \begin{align*}& \mathcal{L}_{\mathrm{KL}} = \sum_{m=1}^{2} \sum_{j=1}^{d_{h}} \left[ \frac{1}{2} \left( \mu_{j,m}^{2} + \sigma_{j,m}^{2} - \log \sigma_{j,m}^{2} - 1 \right) \right]\end{align*}

where $\mu _{j,m}$ and $\sigma _{j,m}^{2}$ denote the mean and variance of the $j$-th dimension of the latent distribution for the $m$-th modality, respectively, and $d_{h}$ is the dimensionality of the latent space.

At the same time, the core objective of the DVAE module is to recover true signals from noisy observations. To this end, we adopt a masking and reconstruction strategy. For each modality $m$, a binary mask $M_{m} \in \{0,1\}^{d_{m}}$ is generated by independent Bernoulli sampling with probability $p_{\mathrm{mask}}$. We extract the masked positions as $\overline{X}_{m}^{\mathrm{mask}} = \bar{X}_{m} \odot M_{m}$, while the original masked values are $X_{m}^{\mathrm{mask}} = X_{m} \odot M_{m}$. The reconstruction loss is measured by mean squared error and is computed *only* on the masked positions: 


(14) \begin{align*}& \mathcal{L}_{\mathrm{recon}} = \sum_{m=1}^{2} \left\| \overline{X}_{m}^{\mathrm{mask}} - X_{m}^{\mathrm{mask}} \right\|_{F}^{2}\end{align*}

where $\overline{X}_{m}^{\mathrm{mask}}$ and $X_{m}^{\mathrm{mask}}$ denote the reconstructed features and the original features at the masked positions for the $m$-th modality, respectively.

Next, we adopt a bidirectional InfoNCE loss as the contrastive learning objective. This design enables the model to automatically discover and align the shared information between the two views while preserving their view-specific characteristics, thereby preventing one view from dominating the learning process. Before similarity calculation, the spatial embedding $Z_{s,i}$ and the expression embedding $Z_{e,i}$ of each cell are first normalized by the $\ell _{2}$ norm. Then, the spatial embedding and the expression embedding are alternately used as queries to retrieve the corresponding positive sample in the other view, while embeddings of other cells are treated as negative samples. The contrastive loss is defined as follows: 


(15) \begin{align*} & \mathcal{L}_{s \rightarrow e} = -\frac{1}{N}\sum_{i=1}^{N} \log \frac{\exp\left(\bar{Z}_{s,i}^{T}\bar{Z}_{e,i}/\tau_{c}\right)} {\sum_{j=1}^{N}\exp\left(\bar{Z}_{s,i}^{T}\bar{Z}_{e,j}/\tau_{c}\right)}\end{align*}

(16) \begin{align*}& \mathcal{L}_{e \rightarrow s} = -\frac{1}{N}\sum_{i=1}^{N} \log \frac{\exp\left(\bar{Z}_{e,i}^{T}\bar{Z}_{s,i}/\tau_{c}\right)} {\sum_{j=1}^{N}\exp\left(\bar{Z}_{e,i}^{T}\bar{Z}_{s,j}/\tau_{c}\right)}\end{align*}

(17) \begin{align*}& \mathcal{L}_{\mathrm{contrast}} = \frac{1}{2}\left(\mathcal{L}_{s \rightarrow e}+\mathcal{L}_{e \rightarrow s}\right)\end{align*}

where $\bar{Z}_{s,i}$ and $\bar{Z}_{e,i}$ denote the normalized embeddings of the $i$th cell in the spatial and expression views, respectively, and $\tau _{c}$ is a temperature parameter controlling the concentration level of the similarity distribution.

However, the contrastive loss mainly focuses on local similarity between paired samples and may overlook global structural information. To enhance the model’s ability to capture the overall data distribution, we further introduce a Deep Graph Infomax (DGI) loss. DGI improves global structure modeling by maximizing the mutual information between node representations and a graph-level summary. Specifically, the graph summary vector is computed as $s_{t}=\sigma \!\left (\frac{1}{N}\sum _{i=1}^{N} Z_{t,i}\right )$, which serves as the average pooled representation of all node embeddings in view $t$. A discriminator is then defined as $D(Z_{t,i}, s_{t})=\sigma \!\left (Z_{t,i}^{T} W_{D} s_{t}\right )$, where $W_{D}$ is a learnable weight matrix. The model is optimized by distinguishing true node embeddings from negative samples generated by randomly shuffling node order. The DGI loss is defined as follows: 


(18) \begin{align*}& \mathcal{L}_{\mathrm{DGI}}=-\frac{1}{N}\sum_{i=1}^{N}\left[\log D(Z_{t,i}, s_{t})+\log\left(1-D(\tilde{Z}_{t,i}, s_{t})\right)\right]\end{align*}

where $\tilde{Z}_{t,i}$ denotes a negative sample generated by randomly shuffling the node order.

In addition, to preserve local spatial continuity in the embedding space, we introduce a spatial regularization loss $\mathcal{L}_{\mathrm{sp}}$, which constrains the representation differences between spatially neighboring nodes. The loss is defined as follows: 


(19) \begin{align*}& \mathcal{L}_{\mathrm{sp}} = \frac{1}{|\mathcal{E}_{s}|} \sum_{(i,j) \in \mathcal{E}_{s}} w_{ij}^{s} \| Z_{i} - Z_{j} \|_{2}^{2}\end{align*}

where $\mathcal{E}_{s}$ is the edge set of the spatial adjacency graph, and $w_{ij}^{s}$ is the adaptive edge weight learned by DGG.

Finally, the overall objective of AGCLD is defined as a weighted combination of all loss terms: 


(20) \begin{align*}& \mathcal{L}_{\mathrm{total}} = \lambda_{k} \mathcal{L}_{\mathrm{KL}} + \lambda_{r} \mathcal{L}_{\mathrm{recon}} + \lambda_{c} \mathcal{L}_{\mathrm{contrast}} + \lambda_{d} \mathcal{L}_{\mathrm{DGI}} + \lambda_{s} \mathcal{L}_{\mathrm{sp}}\end{align*}

where $\lambda _{k}$, $\lambda _{r}$, $\lambda _{c}$, $\lambda _{d}$, and $\lambda _{s}$ are weighting coefficients for the corresponding loss terms.

### Experimental settings

All experiments were conducted on a Windows system equipped with a 2.10 GHz Intel Core i7-12700F processor, 32 GB of memory, and an NVIDIA GeForce RTX 4090 graphics card. To ensure reproducibility, we summarize the hyperparameters used in Supplementary Table S2 and provide a detailed sensitivity analysis of these parameters on five datasets (Supplementary Fig. S4). The AGCLD model has $\sim $380 000–397 000 trainable parameters. Training is completed within 20–42 s, with an average of 36–73 ms per epoch. Peak graphics processing unit (GPU) memory usage ranges from 465 to 985 MB, occupying only about 4% of standard GPU capacity (Supplementary Table S4), which fully demonstrates the computational efficiency of AGCLD.

### Evaluation metrics

According to whether the datasets contain LayerName annotations, we use supervised and unsupervised evaluation metrics to comprehensively assess the performance of the proposed model. For datasets with LayerName annotations, the supervised metrics include Adjusted Rand Index (ARI), Normalized Mutual Information (NMI), Adjusted Mutual Information (AMI), Fowlkes–Mallows Index (FMI), Homogeneity, Completeness, and V-measure. For datasets without LayerName annotations, Moran’s I is used as the unsupervised evaluation metric.

## Results

### Application to the human tonsil dataset

**Figure 2:**
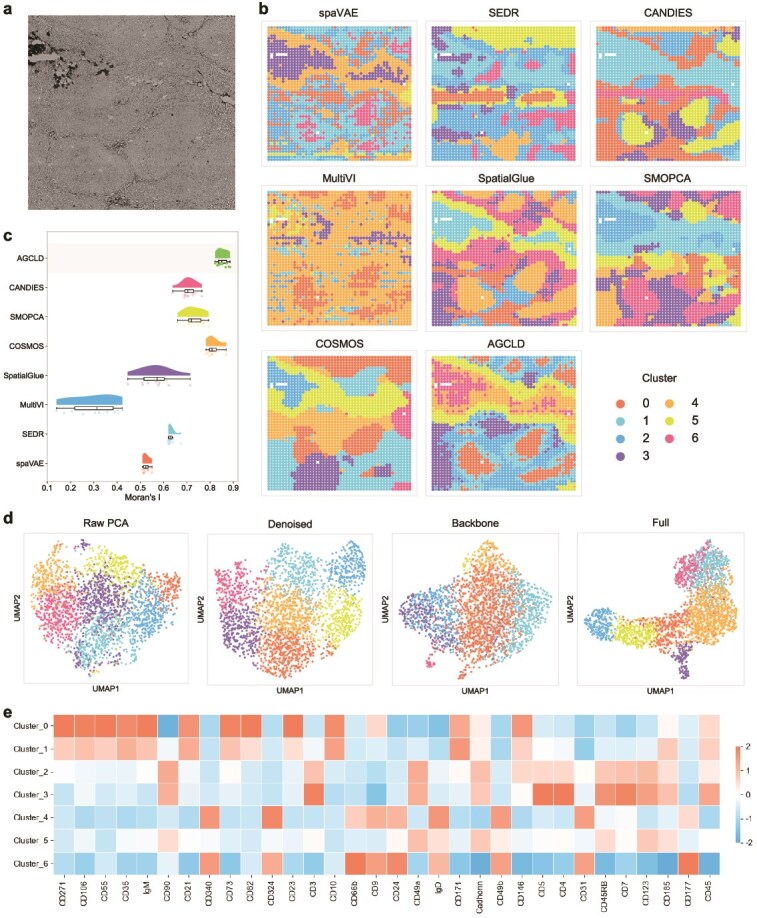
Results on the human tonsil dataset. (a) Histological image of the human tonsil tissue section. (b) Spatial domain identification results of AGCLD and seven comparative methods, including spaVAE, SEDR, MultiVI, SpatialGlue, SMOPCA, COSMOS, and CANDIES. (c) Distribution of Moran’s I scores across repeated experiments for different methods. (d) UMAP visualizations under four settings: Raw PCA, Denoised, Backbone, and Full. (e) Heatmap of differential protein expression across clusters.

We first evaluate the performance of AGCLD on a human tonsil spatial multi-omics dataset. This dataset contains 2492 cells, 984 gene expression features, and 283 surface protein features, covering multiple immune microenvironments, including germinal centers, marginal zones, and T cell regions (Fig. 2a). As shown in Fig. 2b, we compare AGCLD with seven representative methods. Based on the histological annotations provided by Liu *et al.* [9], the spatial domains identified by AGCLD show high consistency with known anatomical structures. Specifically, cluster 0 and cluster 1 correspond to the light and dark zones of the germinal center, cluster 3 represents a T cell-enriched region, cluster 4 corresponds to the crypt epithelium, clusters 2 and 5 are mainly located in the extrafollicular region, and cluster 6 corresponds to peripheral blood cell populations in vascular-related regions. In contrast, the results of MultiVI and spaVAE are relatively scattered and fail to form continuous and stable spatial regions. CANDIES, SEDR, and COSMOS can partially capture local structures but show limitations in delineating clear boundaries and maintaining intra-region consistency. Although SpatialGlue and SMOPCA can recover some tissue patterns, they struggle to achieve fine-granularity partitioning in complex immune microenvironments.

To quantitatively evaluate the spatial domain identification performance, Moran’s I is used to assess the spatial autocorrelation of the results produced by all eight methods. Each method is run 10 times under different random initializations. The results show that AGCLD consistently achieves the highest Moran’s I with low variance, remaining above 0.85 across repeated experiments. Compared with spatial multi-omics methods such as COSMOS, CANDIES, and SMOPCA, AGCLD improves Moran’s I by $\sim $2%–15%, indicating stronger spatial autocorrelation and better stability. In comparison, spaVAE, SEDR, and MultiVI show overall lower scores, suggesting limitations in preserving spatial continuity and structural consistency within tissues. To further investigate the role of the denoising variational autoencoder, Fig. 2d presents uniform manifold approximation and projection (UMAP) visualizations under four settings: Raw PCA (only PCA), Denoised (PCA followed by DVAE), Backbone (PCA followed by the backbone network), and Full (the complete model). Compared with Raw PCA and Backbone, the inclusion of DVAE results in embeddings with improved intra-cluster compactness and inter-cluster separability, demonstrating its effectiveness in reducing noise and enhancing feature representation quality.

To validate the biological relevance of the identified spatial domains, we further analyze the differential protein expression patterns across clusters. As shown in Fig. 2e, marker proteins such as CD23 and CD171 are highly expressed in clusters 0 and 1, consistent with the annotations of the light and dark zones of the germinal center. Cluster 3 shows enrichment of T cell-related proteins, including CD3 and CD4, supporting its identification as a T cell region. Although clusters 2 and 5 are both located in the extrafollicular region, they exhibit distinct protein expression patterns, indicating intra-region heterogeneity. Cluster 6 shows elevated expression of CD31 and CD177, further supporting its annotation as a vascular-related region containing peripheral blood cell populations. These results demonstrate that AGCLD can effectively capture the spatial heterogeneity of the human tonsil immune microenvironment at the molecular level, providing strong biological support for its spatial domain identification results.

### Application to mouse embryonic brain datasets

**Figure 3:**
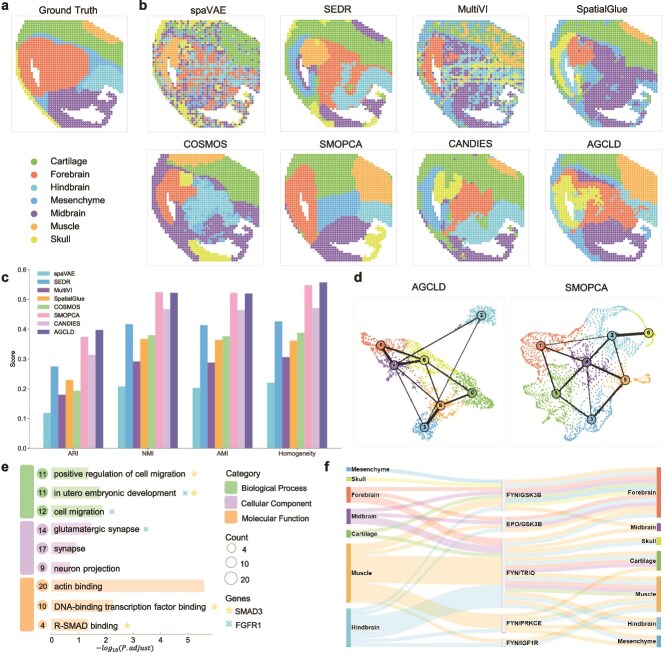
Results on the mouse embryonic E15.5 brain dataset. (a) Ground truth spatial domain annotations of the mouse embryonic E15.5 brain tissue. (b) Spatial domain identification results of AGCLD and seven comparative methods. (c) Quantitative comparison of different methods in terms of ARI, NMI, AMI, and Homogeneity. (d) Visualization of inter-domain structural relationships identified by AGCLD and SMOPCA. (e) Functional enrichment analysis of differentially expressed genes in the Midbrain region. (f) Sankey diagram illustrating cell–cell communication relationships across different tissue regions.

To evaluate the applicability of AGCLD in cross-species scenarios, we further conduct experiments on the mouse embryonic E15.5 brain spatial multi-omics dataset. This dataset contains 1949 cells and covers seven tissue structures (Fig. 3a). Based on this dataset, we compare the spatial domain identification results of AGCLD with seven competing methods (Fig. 3b). The results show that spaVAE and MultiVI produce relatively scattered partitions with noticeable mixing in local regions, failing to capture clear tissue boundaries and spatial continuity. Methods such as SMOPCA, COSMOS, and CANDIES can recover the major spatial structures to some extent, but still exhibit blurred boundaries or incomplete local structures in complex regions. In contrast, the results of AGCLD show better agreement with the ground truth labels in terms of the overall spatial organization.

Furthermore, the quantitative results in Fig. 3c demonstrate that AGCLD achieves an ARI of $\sim $0.40, which is significantly higher than that of SEDR (0.28) and SpatialGlue (0.23). AGCLD also achieves superior performance in terms of NMI and AMI, indicating that it not only better recovers spatial structures but also produces domain partitions more consistent with the ground truth. In addition, from the perspective of inter-domain structural relationships, the spatial domains identified by AGCLD form a more compact and coherent structure. Different domains are connected through reasonable edges to form a stable structural network, whereas SMOPCA, although capable of capturing some major structures, shows more scattered inter-domain connections (Fig. 3d).

Since the Midbrain region of the mouse embryonic E15.5 brain plays a key regulatory role during brain development and is closely associated with neuronal differentiation, regional patterning, and neural circuit formation, we further perform enrichment analysis on 200 differentially expressed genes identified in this region. As shown in Fig. 3e, these genes are mainly enriched in biological processes related to cell migration and embryonic development, and are closely associated with cellular components such as synaptic structures and neuronal projections. At the molecular function level, these genes are mainly involved in cytoskeleton regulation and transcriptional regulation. Notably, SMAD3 and FGFR1 are involved in multiple important pathways, suggesting their potential central roles in the regulation of this region. Based on this, we further construct a Sankey diagram to illustrate cell–cell communication relationships (Fig. 3f). The results indicate that different tissue regions form a complex signaling network through multiple ligand–receptor pairs. In particular, communication axes related to FYN signaling pathways (e.g. FYN/GSK3B and FYN/TRIO) are widely observed across multiple spatial domains and show strong interactions among neural-related regions. These findings demonstrate that AGCLD can not only accurately characterize spatial domain structures but also reveal potential molecular communication mechanisms, thereby providing deeper insights into the relationship between tissue structure and function.

We also apply AGCLD to a mouse embryonic brain dataset generated using the DBiT-seq technique, which enables simultaneous measurement of protein and mRNA expression within the same tissue section to construct spatially resolved multi-omics profiles. Following the preprocessing strategy of Tian *et al.* [30], the data are clustered into six groups. As shown in Supplementary Fig. S2, AGCLD produces smoother spatial domains compared to competing methods, which is further supported by higher Moran’s I scores.

### Application to human lymph node datasets

**Figure 4:**
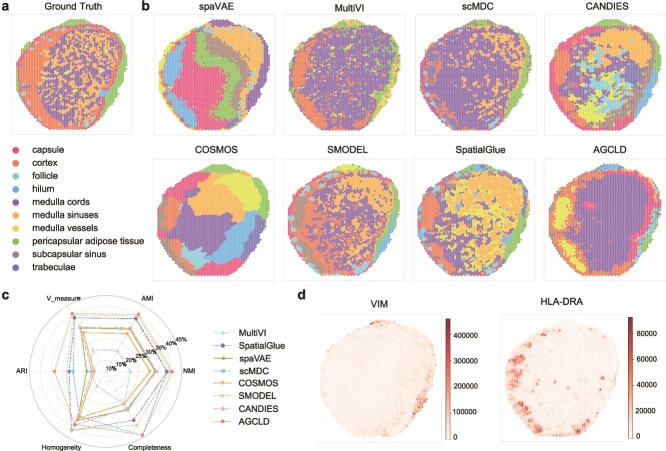
Results on the human lymph node A1 dataset. (a) Ground truth spatial domain annotations of the tissue. (b) Spatial domain identification results of AGCLD and seven comparative methods. (c) Quantitative comparison of different methods in terms of ARI, NMI, AMI, Homogeneity, Completeness, and V-measure. (d) Spatial expression patterns of representative marker proteins, including VIM and HLA-DRA.

To evaluate the performance of AGCLD in analyzing complex tissue structures, we conduct experiments on the human lymph node A1 dataset measured by the 10$\times $ Genomics Visium platform. According to the annotations provided by SpatialGlue, this dataset contains 10 structural types and clearly depicts the continuous tissue organization from the outer capsule to the inner medulla (Fig. 4a). We compare AGCLD with seven representative baseline methods, including spaVAE, scMDC, MultiVI, SpatialGlue, COSMOS, SMODEL, and CANDIES, and evaluate their performance using six widely used clustering metrics (Fig. 4b–c). The results show that AGCLD achieves the highest ARI value (0.33), outperforming the second-best method SMODEL (0.27) and SpatialGlue (0.26) by $\sim $6% and 7%, respectively. In terms of NMI and AMI, which measure the mutual information between clustering results and ground truth labels, AGCLD achieves scores of 0.41 and 0.40, respectively, surpassing SMODEL (0.39 and 0.39) and other competing methods. In addition, AGCLD also performs well on Homogeneity (0.38), Completeness (0.43), and V-measure (0.40), which reflect intra-cluster purity and cluster completeness. These results indicate that AGCLD achieves a better balance between intra-cluster consistency and structural integrity compared to baseline methods such as spaVAE and MultiVI.

We further validate the biological relevance of the identified spatial domains by analyzing the spatial expression patterns of key marker proteins. As shown in Fig. 4d, the expression distributions of these markers are highly consistent with the corresponding anatomical structures. Specifically, VIM shows high expression in medullary cords and stromal regions, reflecting the spatial distribution of mesenchymal and supporting tissues. HLA class II histocompatibility antigen, DR alpha chain (HLA-DRA) is highly enriched in follicular regions, corresponding to immune-active structures such as lymphoid follicles and germinal centers. These results demonstrate that the spatial domains identified by AGCLD are well aligned with both structural organization and functional partitioning, confirming its ability to capture complex tissue architecture. In addition, we further evaluate the cross-slice generalization ability of AGCLD on the human lymph node D1 dataset, with results shown in Supplementary Fig. S3. AGCLD is able to identify spatial domains with high spatial continuity, and the overall partitioning results remain consistent with tissue structures. This further demonstrates the stability and generalization capability of AGCLD across different tissue slices.

## The ablation studies of AGCLD

To validate the contribution of each component in AGCLD, we conducted ablation experiments on five datasets, running each variant five times independently (“w/o” denotes the removal of a specific component), and detailed results are provided in Supplementary Table S3. First, removing the DVAE denoising module reduced the ARI by about 0.2 on both the human lymph node A1 and mouse embryonic E15.5 brain datasets, indicating that modality-specific denoising is a key prerequisite for obtaining high-quality latent representations under the noisy and sparse conditions typical of spatial multi-omics data. Second, removing the DGG or the cross-modality fusion module led to clear performance drops, reflecting the importance of adaptive neighborhood modeling and cross-modal complementary information in clustering discrimination. Furthermore, removing the dual-graph contrastive objective or the adaptive fusion mechanism also resulted in a consistent decrease in Moran’s I on the three datasets without ground truth annotations, suggesting that contrastive alignment between graph branches and data-driven modality weighting directly affects the delineation of spatial domains.

In addition, we conducted three information-theoretic experiments on the human lymph node A1 dataset to further evaluate the internal representations of AGCLD. As shown in Supplementary Fig. S5, the InfoNCE MI lower bound remained comparable across all configurations (4.51–4.67). This indicates that even after removing some components, the model still maintains adequate cross-view alignment. However, in the mutual information (Î) experiments, removal of DGI or DVAE reduced this metric to 0.085 and 0.066 [47]. These results suggest that both the node graph MI objective and the denoising module are critical for preserving domain-discriminative information. Meanwhile, removing DGI in the spatial coherence (Moran’s I) experiments caused a sharp decline from 0.694 to 0.207. In summary, each component is indispensable for preserving domain-discriminative information and maintaining spatial coherence. This ensures robust and stable performance across different datasets.

## Conclusion

In this work, we propose AGCLD to address key limitations in existing spatial multi-omics analysis methods, including insufficient denoising and reliance on fixed graph structures. The proposed model improves feature representation quality through modality-specific denoising and employs a DGG to adaptively model cell–cell neighborhood relationships, enabling more flexible characterization of spatial heterogeneity within tissues. In addition, the dual-graph attention encoder, combined with a contrastive learning strategy, effectively aligns spatial information and expression features, further enhancing the consistency of the learned embeddings. Extensive experiments on multiple real spatial multi-omics datasets demonstrate that AGCLD outperforms existing methods in spatial domain identification tasks, while maintaining strong stability and robustness under complex data conditions.

The current model is mainly designed for two-modality data. Although systematic validation on datasets with three or more concurrently measured omics modalities remains to be conducted, AGCLD can be naturally extended to the integration of three or more omics modalities through its modular architecture. In addition, under extremely high sparsity or strong batch effects, the representation learning capability of the model may still be affected. In future work, we plan to investigate more general multi-modal fusion strategies and incorporate histological image information to further improve the modeling of spatial structures and molecular expression relationships.

**Key Points**

- We propose adaptive graph contrastive learning method with denoising (AGCLD), a spatial multi-omics integration framework that combines denoising variational autoencoder, adaptive graph learning, and contrastive learning for spatial domain identification.
- AGCLD introduces a differentiable graph generator to adaptively construct spatial and expression graphs, enabling cell-specific neighborhood modeling and overcoming the limitations of predefined graph structures.
- A dual-graph attention encoder with contrastive learning is designed to align spatial information and molecular features, improving representation consistency and discriminative capability.
- Extensive experiments on multiple spatial multi-omics datasets demonstrate that AGCLD achieves strong performance and stability in spatial domain identification tasks.

## Supplementary Material

Supplementary_materials_bbag385

## Data Availability

All spatial multi-omics datasets supporting this study are publicly available. The human tonsil (spatial-CITE-seq), mouse embryonic E15.5 brain (MISAR-seq), and mouse embryonic brain (DBiT-seq) datasets are available at https://figshare.com/articles/dataset/Spatial_genomics_datasets/21623148/5. The human lymph node A1 and D1 datasets measured by the 10x Genomics Visium platform are available at https://zenodo.org/records/10362607. Detailed information about these datasets is provided in Supplementary Table S1. The source code could be accessed and downloaded from https://github.com/lwyaq/AGCLD
