# Supplementary material for: AGCLD: an adaptive graph contrastive learning method with denoising for spatial domain identification
Source: Brief Bioinform. 2026 Jul 14;27(4):bbag385. doi: 10.1093/bib/bbag385 (PMC13367445; doi:10.1093/bib/bbag385)
Supplement: Supplementary_materials_bbag385 [file supplementary_materials_bbag385.pdf]

Supplementary materials for

AGCLD: An Adaptive Graph Contrastive Learning Method with Denoising for Spatial Domain Identification

Yating Li[1,2,3], Xinyue Yu[1,2,3], Hao Zhang[1,2,3], Hao Lin[4], Bo Liu[1,2,3,*], Haixia Long[1,2,3,*]

[1] School of Artificial Intelligence, Hainan Normal University, Haikou 571158, China.

[2] Key Laboratory of Data Science and Smart Education, Ministry of Education, Hainan Normal University, Haikou 571158, China.

[3] Haikou Key Laboratory of Intelligent Analysis and Secure Sharing of Tropical Biodiversity Data, Hainan Normal University, Haikou 571158, China.

[4] School of Life Science and Technology, University of Electronic Science and Technology of China, Chengdu 610054, China.

[*] Correspondence: Bo Liu, Haixia Long.
Bo Liu: boliu@hainnu.edu.cn; Haixia Long: lhx@hainnu.edu.cn

Supplementary Table S1 Summary of spatial multi-omics datasets used in this study

Platform	Dataset	Cell	RNA	Protein	ATAC	LayerName
spatial-CITE-seq	human tonsil	2492	984	283	–	No
MISAR-seq	mouse embryonic E15.5 brain	1949	21808	–	47287	Yes
DBiT-seq	mouse embryo brain	1789	284	22	–	No
10x Genomics Visium	human lymph node A1	3484	18085	31	–	Yes
10x Genomics Visium	human lymph node D1	3359	18085	31	–	No

Supplementary Table S2 Hyperparameter settings used in this study

Dataset	p_{mask}	λ_k	λ_r	λ_c	λ_d	λ_s	head_r
human tonsil	0.30	0.01	2.0	1.5	0.8	0.2	2
mouse embryonic E15.5 brain	0.05	0.10	0.5	1.0	1.5	0.5	2
mouse embryo brain	0.30	0.01	2.0	1.5	0.8	0.2	2
human lymph node A1	0.50	0.01	2.0	1.5	0.8	0.2	2
human lymph node D1	0.30	0.01	2.0	1.5	0.8	0.2	2

Supplementary Table S3 Ablation study results

Method	human lymph node A1	mouse embryonic E15.5 brain	human tonsil	mouse embryo brain	human lymph node D1
	ARI		Moran's I		
w/o DVAE	0.1466±0.0127	0.2071±0.0177	0.8036±0.0818	0.5839±0.1062	0.2644±0.0623
w/o Cross-Modality Fusion	0.2388±0.0262	0.1941±0.0128	0.8161±0.0518	0.5493±0.0616	0.3486±0.0564
w/o DGG	0.2663±0.0203	0.2112±0.0210	0.6440±0.0954	0.5719±0.0220	0.4053±0.1348
w/o Dual-Graph CL	0.1792±0.0151	0.2829±0.0456	0.8137±0.0301	0.7739±0.0646	0.5762±0.0589
w/o Adaptive Fusion	0.2145±0.0196	0.2460±0.0244	0.7712±0.0551	0.4995±0.0545	0.4600±0.0674
**AGCLD**	**0.3285±0.0170**	**0.3980±0.0144**	**0.8542±0.0185**	**0.8781±0.0221**	**0.7953±0.0093**

Supplementary Table S4 Runtime and memory usage of AGCLD on five spatial multi-omics datasets.

Dataset	Cell	Number of parameters	Train time (s)	Time/epoch (ms)	Peak GPU (MB)	Peak RAM (MB)
human tonsil	2492	397K	19.9	35.5±12.8	676	2.7
human lymph node A1	3484	384K	36.3	47.3±19.4	985	3.1
human lymph node D1	3359	384K	41.4	73.2±4.1	942	2.9
mouse embryo brain	1789	379K	29.1	72.0±3.4	465	1.8
mouse embryonic E15.5 brain	1949	397K	26.4	71.9±3.0	510	1.9

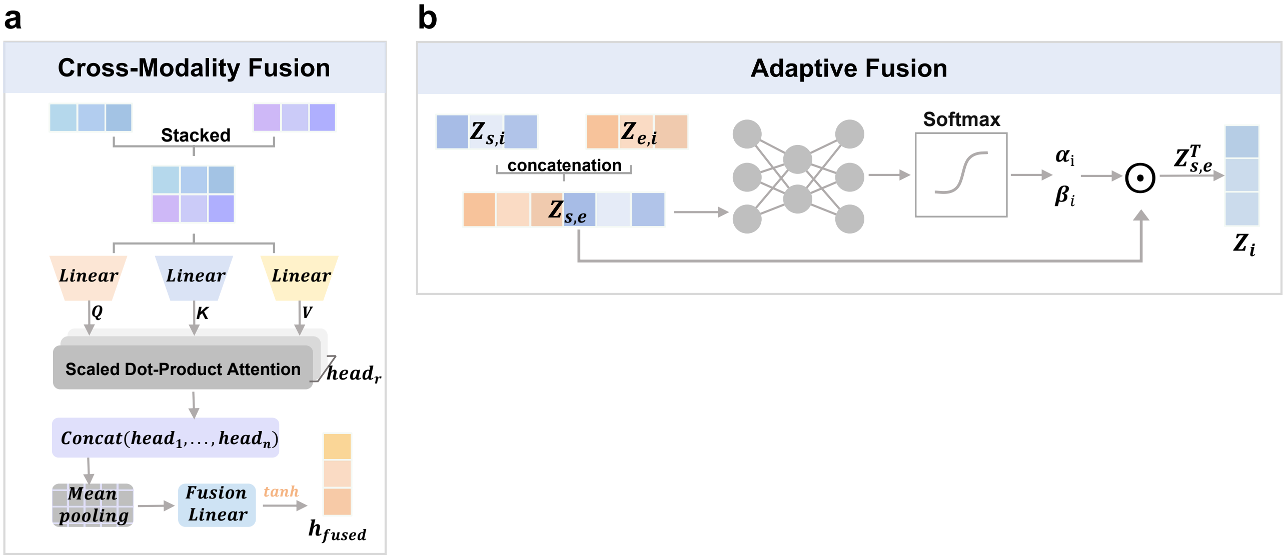

Supplementary Fig. S1 Supplementary model detail figures. (a) Cross-Modality Fusion module. (b) Adaptive Fusion module.

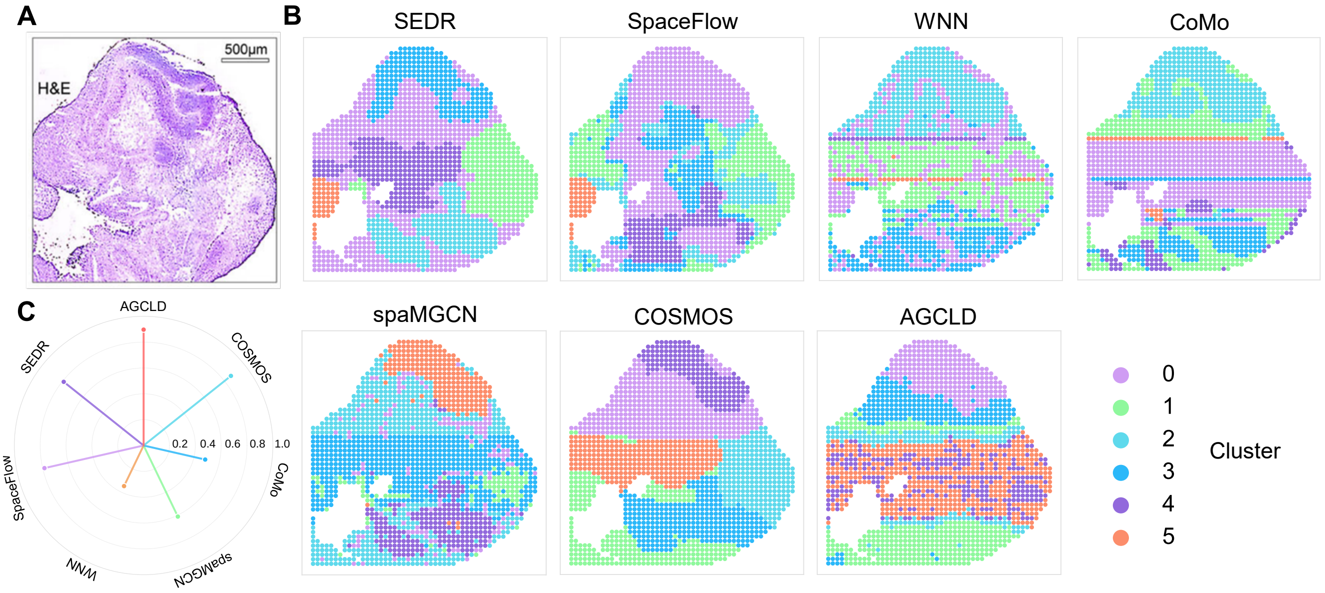

Supplementary Fig. S2 Experimental results on the mouse embryo brain dataset. (A) H&E stained section of the mouse embryonic brain. (B) Spatial domain visualizations using seven different methods. (C) Moran's I values for the seven methods, illustrating the spatial autocorrelation of the detected domains.

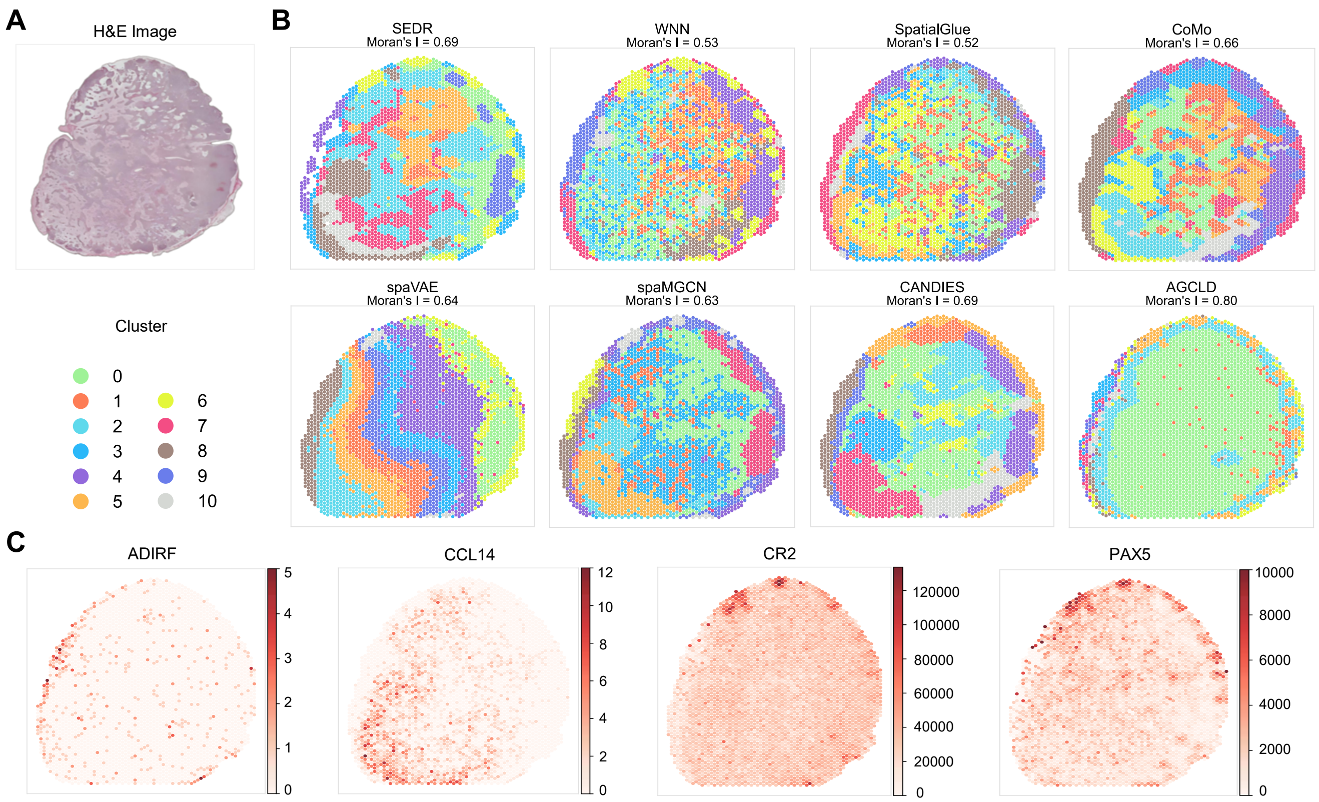

Supplementary Fig. S3 Experimental results on the human lymph node D1 dataset. (A) H&E staining of the human lymph node D1 tissue section. (B) Spatial domain visualization results of eight methods, with the spatial autocorrelation of the detected domains quantified by the Moran's I metric. (C) Spatial expression density heatmaps of two key marker genes and two key marker proteins in the dataset.

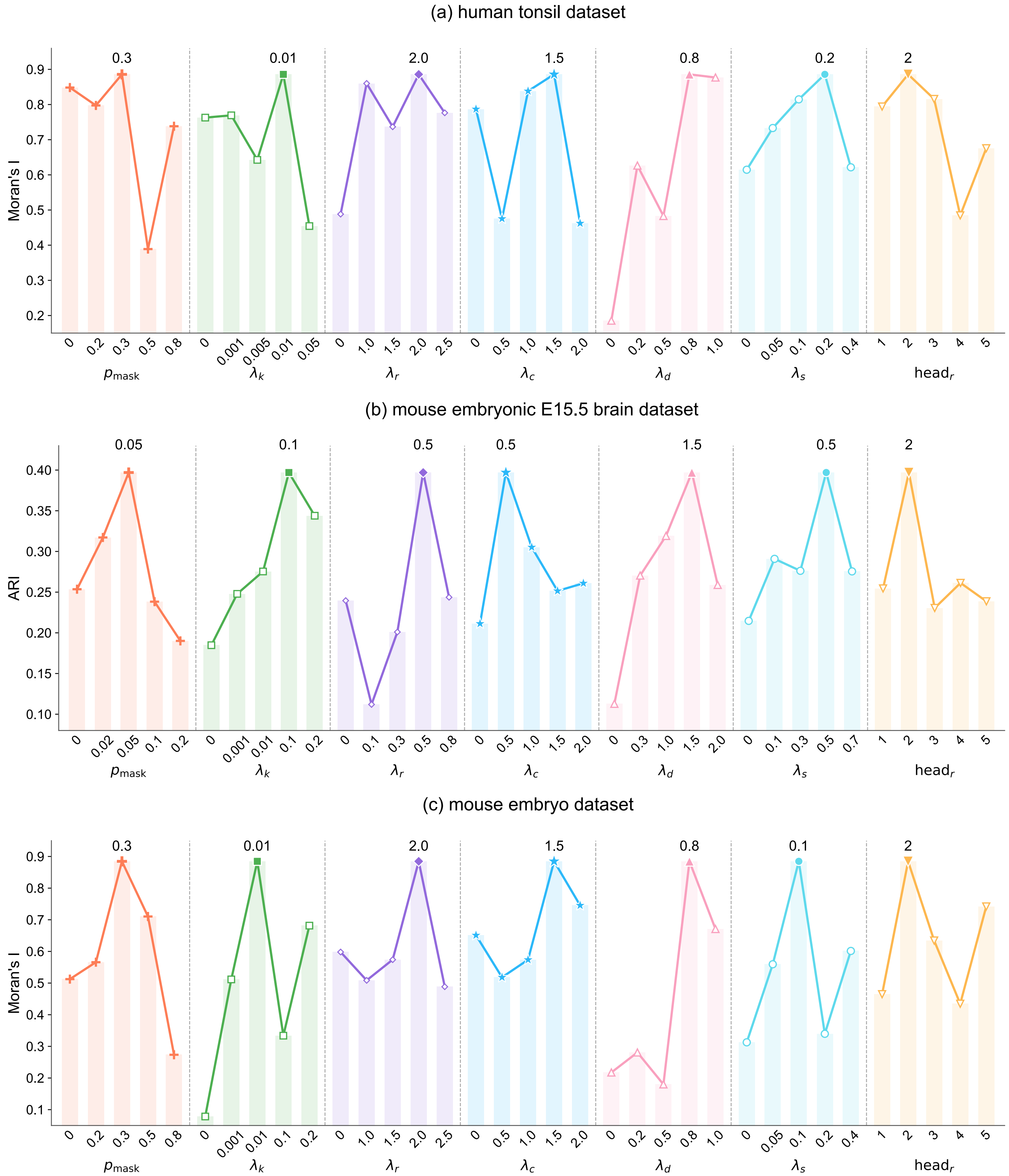
(a) human tonsil dataset
Moran's I
0.3
0.01
2.0
1.5
0.8
0.2
2
0.2 0.3 0.4 0.5 0.6 0.7 0.8 0.9
0 0.2 0.3 0.5 0.8
0 0.001 0.005 0.01 0.05
0 1.0 1.5 2.0 2.5
0 0.5 1.0 1.5 2.0
0 0.2 0.5 0.8 1.0
0 0.05 0.1 0.2 0.4
1 2 3 4 5
p_{mask}
λ_k
λ_r
λ_c
λ_d
λ_s
$head_r$
(b) mouse embryonic E15.5 brain dataset
ARI
0.05
0.1
0.5
0.5
1.5
0.5
2
0.10 0.15 0.20 0.25 0.30 0.35 0.40
0 0.02 0.05 0.1 0.2
0 0.001 0.01 0.1 0.2
0 0.1 0.3 0.5 0.8
0 0.5 1.0 1.5 2.0
0 0.3 1.0 1.5 2.0
0 0.1 0.3 0.5 0.7
1 2 3 4 5
p_{mask}
λ_k
λ_r
λ_c
λ_d
λ_s
$head_r$
(c) mouse embryo dataset
Moran's I
0.3
0.01
2.0
1.5
0.8
0.1
2
0.1 0.2 0.3 0.4 0.5 0.6 0.7 0.8 0.9
0 0.2 0.3 0.5 0.8
0 0.001 0.01 0.1 0.2
0 1.0 1.5 2.0 2.5
0 0.5 1.0 1.5 2.0
0 0.2 0.5 0.8 1.0
0 0.05 0.1 0.2 0.4
1 2 3 4 5
p_{mask}
λ_k
λ_r
λ_c
λ_d
λ_s
$head_r$

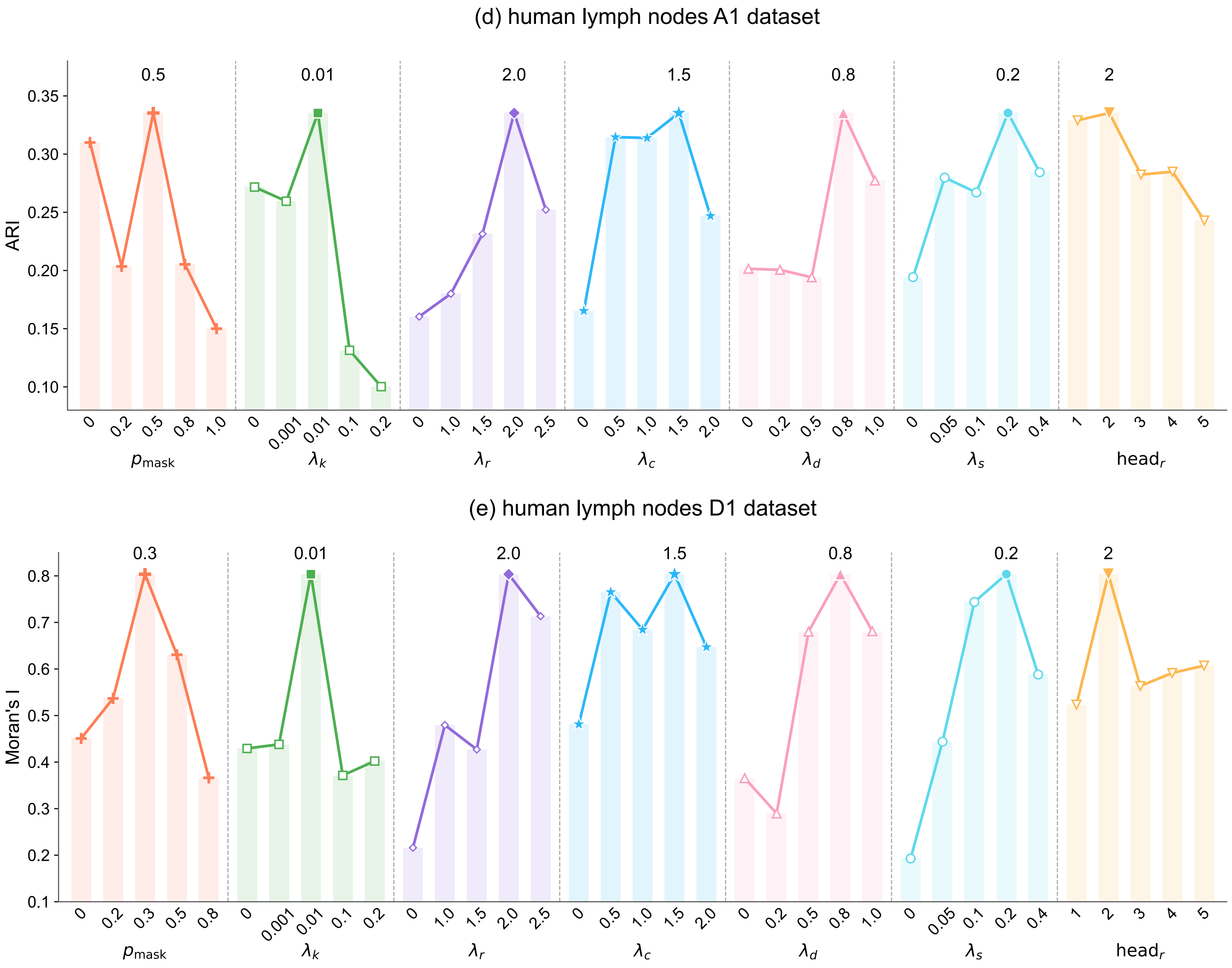

Supplementary Fig. S4 Hyperparameter ablation experiments across five datasets.

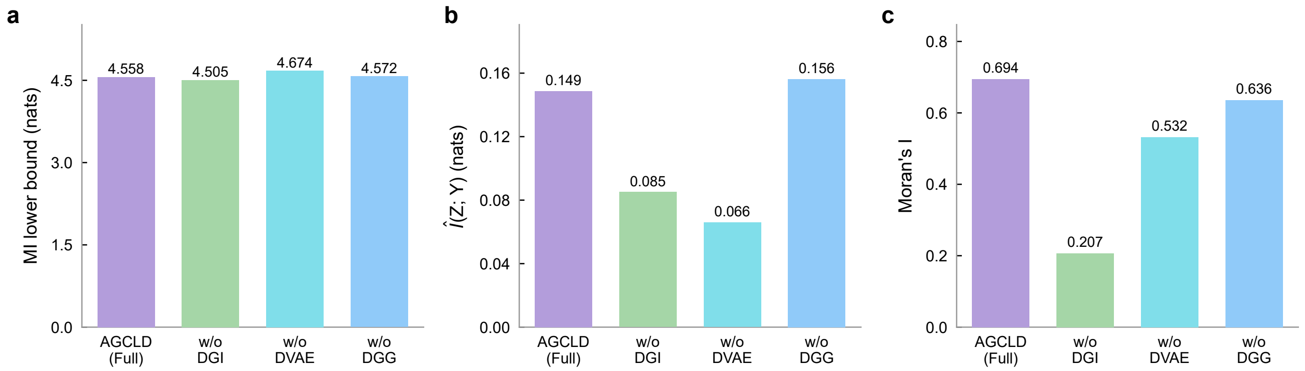

Supplementary Fig. S5 Information-theoretic validation on the Human Lymph Node A1 dataset. (a) Final InfoNCE mutual information lower bound for the full AGCLD model and three ablation variants. (b) Mutual information $\hat{I}$ between the learned embedding and ground-truth domain labels, estimated using a k nearest neighbour mutual information estimator. (c) Moran's I spatial autocorrelation of the predicted domain partitions.
